# The homogeneity and heterogeneity of occurrence, characteristics, and prognosis in hepatocellular carcinoma patients with synchronous and metachronous bone metastasis

**DOI:** 10.7150/jca.65308

**Published:** 2022-01-01

**Authors:** Yanting Zhang, Yao Xu, Wenjuan Ma, Haixiao Wu, Guijun Xu, Vladimir P. Chekhonin, Karl Peltzer, Xin Wang, Guowen Wang, Chao Zhang

**Affiliations:** 1 Tianjin Medical University Cancer Institute and Hospital, National Clinical Research Center for Cancer, Key Laboratory of Cancer Prevention and Therapy, Tianjin's Clinical Research Center for Cancer, Tianjin, China.; 2 The Sino-Russian Joint Research Center for Bone Metastasis in Malignant Tumor, Tianjin, China.; 3 Department of Orthopedics, Tianjin Hospital, Tianjin University, Tianjin, China.; 4 Department of Basic and Applied Neurobiology, Federal Medical Research Center for Psychiatry and Narcology, Moscow, Russian Federation.; 5 Department of Psychology, University of the Free State, Turfloop, South Africa.; 6 Department of Epidemiology and Biostatistics, West China School of Public Health, Sichuan University, South Renmin Road, Wuhou District, Chengdu, China.

**Keywords:** hepatocellular carcinoma, synchronous bone metastasis, metachronous bone metastasis, risk, prognosis

## Abstract

**Purpose:** Based on the one of the largest hepatocellular carcinoma (HCC) population with bone metastasis (BM) from the single center in Tianjin, China, the present study aimed to investigate the risk and survival of synchronous bone metastasis (sBM) and metachronous bone metastasis (mBM) in HCC, and to reveal characteristics and related factors of HCC patients with bone metastasis.

**Methods:** HCC patients with bone metastasis between 2009 and 2017 from Tianjin Medical University Cancer Institute & Hospital, Tianjin, China, were involved. Chi-square test/ Fisher's exact test and Logistic regression were used to estimate the risk factors of bone metastasis in HCC. Kaplan-Meier method was used to estimate the survival of HCC patients, and the Log-rank test was used to analyze the survival of HCC patients. The prognostic factors of HCC patients with BM were identified via Kaplan-Meier method and multivariable COX regression model.

**Results:** Among 4421 HCC patients, 128 patients with BM were identified. Of the 128 patients with BM, 77 patients (60.16%) were with sBM and 51 patients (39.84%) were with mBM. The incidence of sBM in HCC was 1.74% at initial diagnosis. The most common metastatic site of sBM was rib, followed by lumbar, thoracic, and sacral. The median latency time from HCC diagnosis to mBM was six months. The most common site of mBM was thoracic, followed by lumbar, sacral and rib. Alcohol-drinking history (*P*=0.027), numbers (*P*=0.023) and size (*P*=0.008) of intrahepatic tumor, lymph node metastasis (*P*<0.001), serum ALP (*P*=0.004) and HGB (*P*=0.004) level were found to be correlated with the occurrence of BM. The overall survival between non-BM and BM were statistically different (*P*=0.028).

**Conclusion:** The incidence of sBM in HCC was 1.74% at initial diagnosis. The median latency time from HCC diagnosis to mBM was 6 months. The characteristics between occurrence and prognosis showed significant difference between sBM and mBM. Early identification of high-risk BM population was essential for the improvement of both quality of life and prognosis. The revealed related factors can potentially guide sBM and mBM identification and early diagnosis in HCC.

## Introduction

Hepatocellular carcinoma (HCC) was the fifth common cancer in the world, and it was the second most fatal tumor 1, 2. It was globally estimated that 250,000 people died of HCC each year. Most HCC patients were diagnosed at the advanced stage, thus HCC patients usually showed the poor prognosis. The 5-year survival rate of HCC patients was reported to be less than 20% 3, 4. Bone metastasis (BM) was accepted as one of the factors that impeded both the survival and quality of life for HCC patients. A total of 25% HCC patients showed BM at initial diagnosis or at their later course. BM rarely resulted in the death of HCC patients, but the pain and dysfunction caused by BM significantly reduced the patients' quality of life. To improve the quality of life and the survival of patients, the early diagnosis of BM in HCC is important.

The most metastatic sites in HCC patients with BM were reported to be spine, pelvis, and ribs, of which spinal metastasis accounts for 40% 5, 6. Skeletal related events (SREs), resulted by BM, were defined as severe pain, pathological fractures, malignant hypercalcemia, spinal cord compression, and other neurological compression syndrome 7. SREs dramatically increased the analgesic use and reduced the quality of patients' life. As reported in the previous studies, once BM occurred in HCC patients, the 2-year survival was less than 5% 8. According to the occurrence time of BM, BM is divided into synchronous BM (sBM) and metachronous BM (mBM). SBM was defined as the occurrence of BM at HCC diagnosis, while mBM was defined as the occurrence of BM at patients' later course. Considering different diagnosis and treatment procedures, clinicopathological characteristics and prognostic outcomes of sBM and mBM in HCC can be found. However, constricted by the sample size, seldom study reported the difference between sBM and mBM in HCC. In HCC patients with sBM, the proper diagnosis of sBM can guide the prognostic prediction and the individualized treatment generation. While in the patients with mBM, timely screening of BM is of significance on guiding bone targeting therapy. Thus, the study looking into sBM and mBM in HCC is warrant.

Based on a single center population, we systematically evaluated the difference on occurrence, progression and prognosis between sBM and mBM patients with HCC. The results in the present study can potentially guide individualized BM screening and treatment for BM in HCC.

## Patients and methods

**Figure 1:**
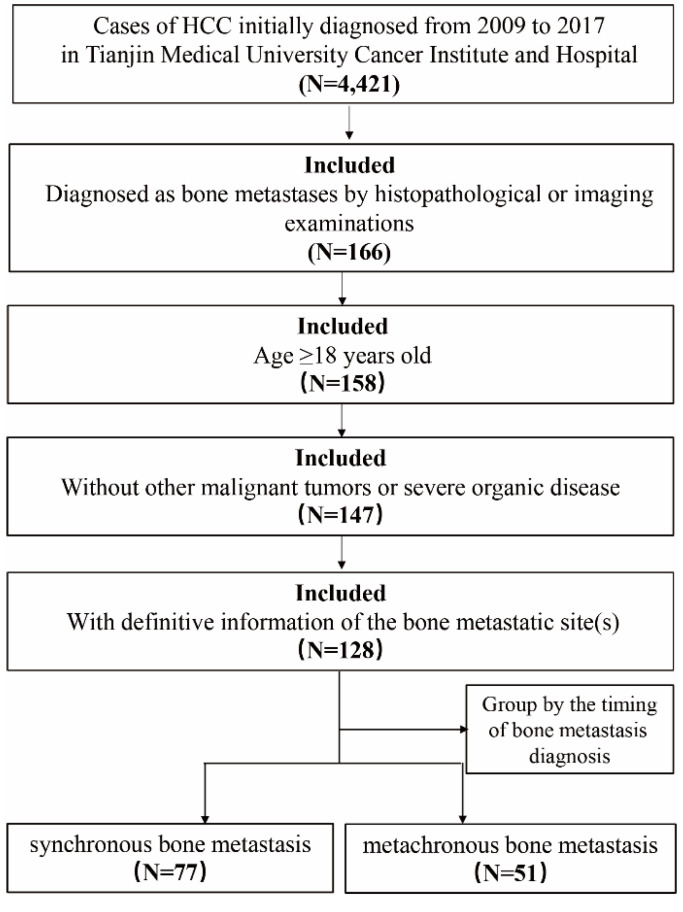
Flowchart of the patient selection process in the present study.

Patients who were diagnosed with HCC between January 2009 and December 2017 in Tianjin Medical University Cancer Institute & Hospital (Tianjin, China), were retrospectively identified. The inclusion criteria were as follows: 1. over the age of 18; 2. diagnosed as BM by histopathological and/or imaging examinations such as standard X-rays, whole-body bone scans, computed tomography (CT), magnetic resonance imaging (MRI) and positron emission tomography-computed tomography (PET-CT); 3. without other malignant tumors or severe organic disease; 4. with definitive information of the bone metastatic site(s). HCC patients with non-BM were randomly drawn from the same period. In order to compare the characteristics of patients with bone metastasis and those without bone metastasis, we performed 1 : 2 population matching to determine the sample size of non-BM group. In our study, mBM was defined when HCC was diagnosed prior to the BM diagnosis more than 3 months, otherwise patients were considered to be the part of the sBM (Figure 1). The overall survival (OS) was defined as the time from HCC diagnosis to cancer-associated death or to the end of follow up. The time of follow-up was performed from HCC diagnosis to death or January 2020. Latency time in HCC patients with mBM referred to the time from HCC diagnosis to BM occurrence, and survival after BM was defined as the time from BM diagnosis to death or the end of follow-up. This study was approved by the Ethics Committee of Tianjin Medical University Cancer Institute and Hospital, Tianjin, China.

Patient demographics and tumor variables were collected. Patient demographic variables, including gender, age at diagnosis, ABO blood type and related medical history. A series of tumor characteristics were collected, including number, size, serum ALP and AFP, blood cell counts, and metastatic site (bone, lymph node and other sites), as well as treatment modalities, such as surgical type. Information on both the number and specific sites of BM in HCC were collected.

### Statistical analyses

All statistical analyses were performed using SPSS version 21.0 (IBM, Armonk, NY). We used method of multiple imputation to handle missing data. The Chi-square test or Fisher's exact test was used to make the consistent of the baseline characteristics of HCC patients with/without BM. Variables that had an association with a P <0.2 based on univariate analysis were included in multivariate logistic regression model. OS was assessed with the Kaplan-Meier method, and the Log-rank test was used to study subgroups. Those variables with *P* value < 0.2 in the Kaplan-Meier analysis were included in the multivariate COX regression analysis. Factors in which *P* < 0.05 based on COX regression analyses were identified as the independent prognostic variables. And we used Cox regression analysis to calculate the hazard ratio (HR) and 95% confidence intervals (95% CI). It was considered statistically significant if *P* <0.05.

## Results

### Characteristics of study population

HCC patients who were diagnosed with HCC between January 2009 and December 2017 in Tianjin Medical University Cancer Institute and Hospital (Tianjin, China), were retrospectively identified. A total of 4421 patients were diagnosed as HCC in our hospital, including 166 patients with BM. According to the inclusion and exclusion criteria, a total of 128 patients were included. There were 77 patients with sBM, including 66 male patients (85.7%), 11 female patients (14.3%), and aged ≤40, 41-60, and ≥61 years old patients were 3 (3.9%), 49 (63.6%), 25 (32.5%) respectively; There were 51 patients with mBM, 48 male patients (94.1%), 3 female patients (5.9%), and 3 (5.9%), 28 (54.9%), and 20 (39.2%) patients with age ≤40, 41-60, and ≥61, respectively. The median age of patients was 57 years (range 49-63) in sBM group and 56 years (range 51-64) in mBM group respectively. In addition, 263 HCC patients with non-BM were randomly selected. The population distribution of HCC patients was shown in Table 1.

### Metastatic sites

In 128 HCC patients with BM, a total of 297 metastatic sites of bone were found. A total of 201 sites were found in sBM while 96 sites were found in mBM. The most common metastatic site in sBM was ribs (41 sites), followed by lumbar (34 sites), thoracic (31 sites), and sacral (24 sites). The most common metastatic site in mBM was thoracic (23 sites), followed by lumbar (21 sites), sacral (15 sites) and ribs (10 sites). Together, the most common metastatic site was trunk (231sites, 77.8%), followed by upper limb (38 sites, 12.8%), lower limb (16 sites, 5.4%), and skull (12 sites, 4.0%).

**Figure 2:**
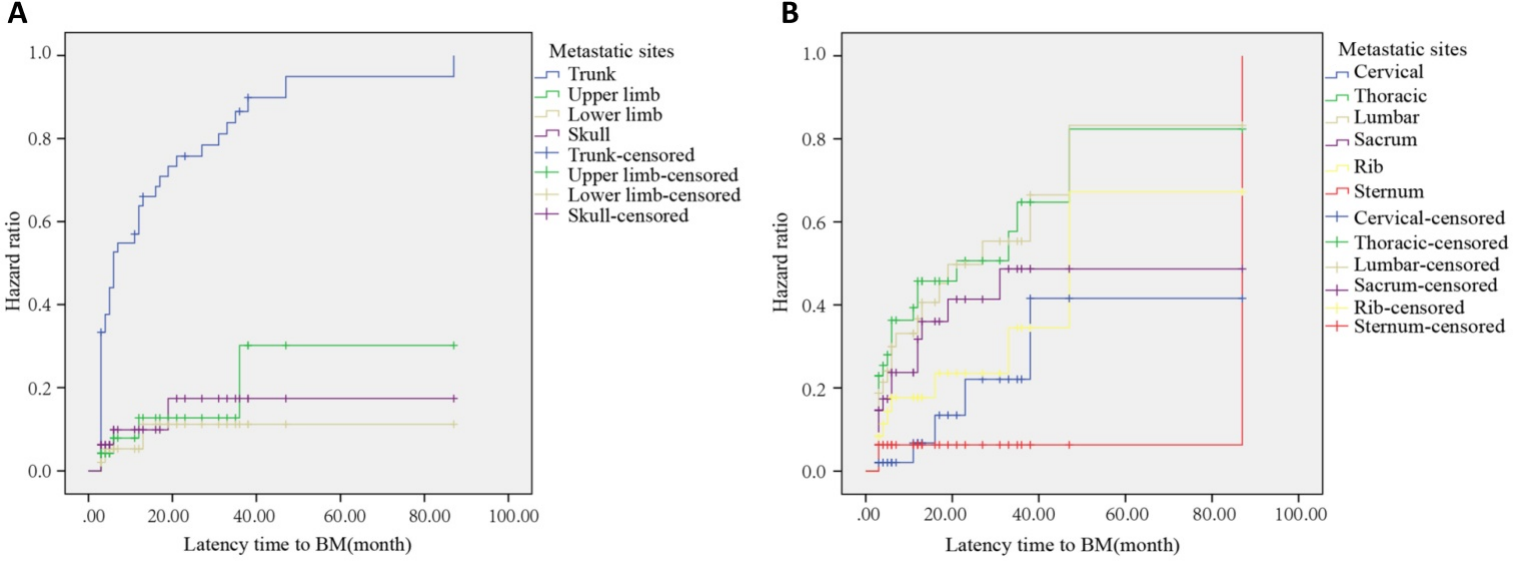
(**A**) Hazard curve of whole body bone metastases after HCC diagnosis. (**B**) Hazard curve of trunk metastases after HCC diagnosis.

In HCC patients with mBM, the most common bone metastatic site at 6, 12, and 24 months after HCC diagnosis was the trunk, with the incidences of 52.1%, 62.5%, and 72.9%, respectively (Figure 2A). After Log-Rank test, the difference between trunk and non-trunk in the incidence of BM was statistically significant (*P*<0.001). Among all metastatic sites, the most common metastatic site was the thoracic. The risk of metastasis at 6, 12, and 24 months after HCC diagnosis was 69.6%, 82.6%, and 87.0%, respectively (Figure 2B). At 6, 12 and 24 months after HCC diagnosis, the commonly found bone metastatic sites were thoracic, thoracic, and sacral, respectively. Among the 128 HCC patients with BM, 101 patients were found to be with bone-only metastases, and 27 patients were found to be with multi organs metastasis. The most common metastasis site was lung (53.0%), while the least was brain (2.9%) (Supplementary Table S1).

### Risk factors of BM in HCC

**Figure 3:**
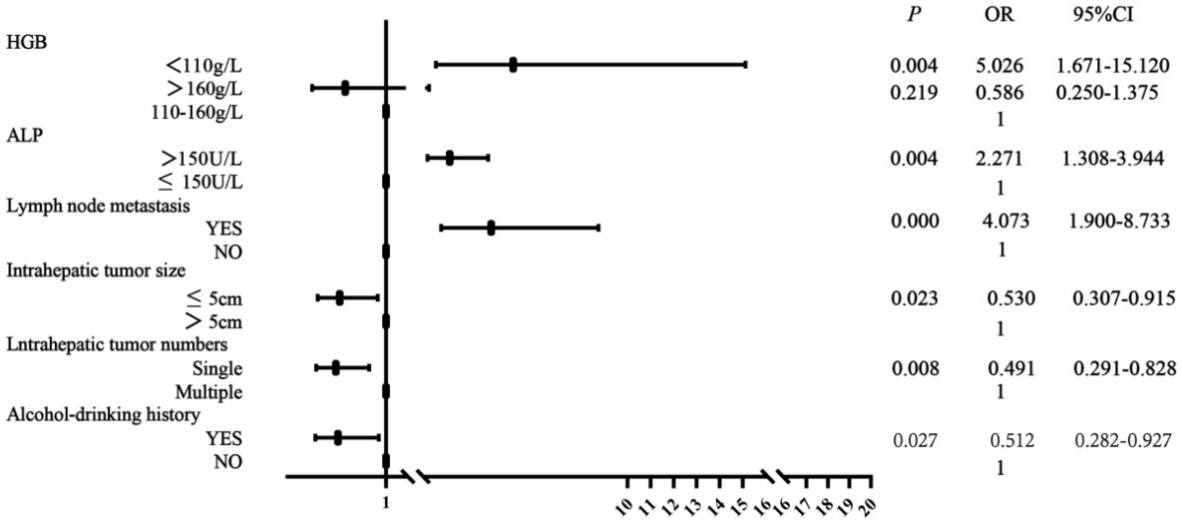
Forest plot of risk factors for BM occurrence. OR (odds ratio), 95%CI (confidence interval).

As shown in Table 2, univariate and multivariate analyses of risk factors for HCC patients with BM showed that there was no significant difference in gender, age, ABO blood type, smoking history, family history of cancer, antiviral history, vascular tumor thrombus, intrahepatic metastasis, extraosseous metastases, serum AFP, HBsAg, WBC and PLT level between BM patients and non-BM patients. There were significant differences in alcohol-drinking history, numbers and size of intrahepatic tumor, lymph node metastasis, serum ALP and HGB level. Multivariate analysis showed that HGB level <110 g/L (*P*=0.004, OR=5.026), serum ALP >150 U/L (*P*=0.004, OR=2.271), presence of lymph node metastasis (*P*<0.001, OR=4.073) were correlated with BM occurrence in HCC. Solitary intrahepatic tumor (*P*=0.008, OR=0.491), intrahepatic tumor size ≤5 cm (*P*=0.023, OR=0.530) and presence of alcohol-drinking history were protective factors for BM occurrence in HCC. Figure 3 showed forest plot of multivariate analysis for risk factors associated with BM occurrence.

After univariate and multivariate analyses, intrahepatic tumor size, lymph node metastasis, serum ALP and HGB levels were related with sBM occurrence in HCC. Gender, ABO blood type, alcohol-drinking history, intrahepatic tumor numbers, extraosseous metastases, HGB and PLT level were relevant to mBM occurrence. The related factors of HCC patients with sBM and mBM were illustrated in Table 3 and Table 4, respectively. Patients with lymph node metastasis, serum ALP >150 U/L, and HGB <110 g/L were more likely to occur sBM compared with non-BM, while intrahepatic tumor size ≤5 cm was the protective factor for sBM occurrence. Compared with non-BM, male gender, AB blood type, presence of extraosseous metastases, HGB <110 g/L and PLT <100×10^9^/L were risk factors for mBM. Patients with solitary intrahepatic tumor and alcohol-drinking history were less likely to occur mBM. Forest plots of relative factors associated with sBM and mBM occurrence were illustrated in Supplementary Figure S1 and Supplementary Figure S2.

### Survival estimation in HCC patients with BM

**Figure 4:**
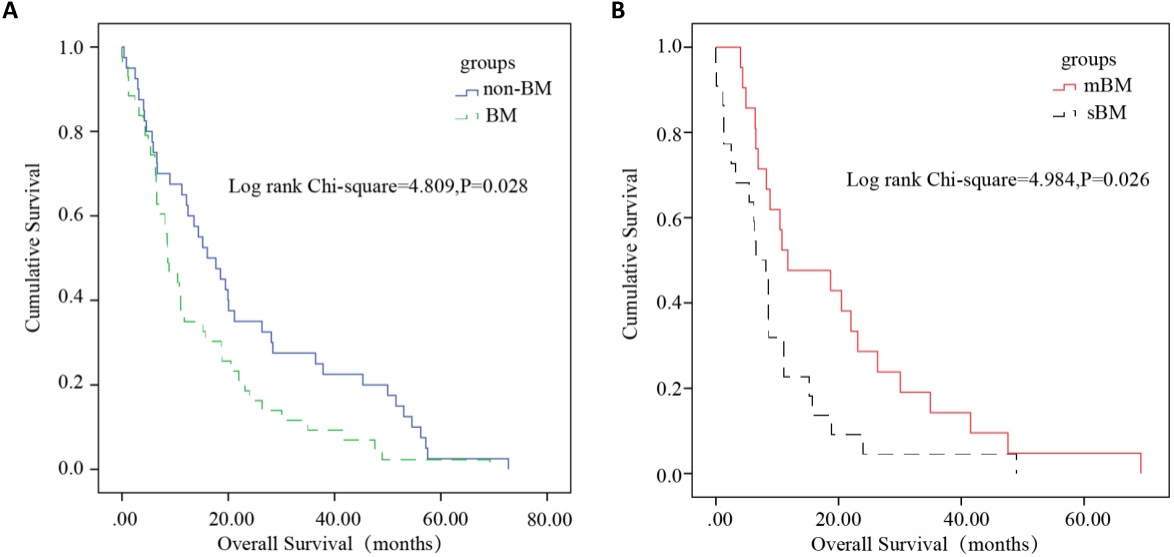
** Kaplan-Meier overall survival analysis of HCC patients.** *P* < 0.05 was considered to be statistically significant. **(A)** Survival analysis was performed between non-BM and BM. The survival of HCC patients with BM was significantly worse (*P*=0.028). **(B)** Survival analysis was performed between sBM and mBM. The survival of HCC patients with sBM was significantly worse than mBM (*P*=0.026).

To the end of the follow-up, a total of 83 patients completed whole follow-up (40 non-BM cases/43 BM cases). The median survival of HCC patients in the non-BM and BM groups were 16.07 months and 8.61 months, respectively. Among 43 BM patients with complete follow-up information (21 mBM cases/22 sBM cases), the median survival of patients in the sBM and mBM groups were 6.51 months and 11.73 months, respectively. After Log-rank test, the differences of overall survival between non-BM and BM (*P*=0.028), sBM and mBM (*P*=0.026) were statistically significant (Figure 4). For patients with mBM, the distribution of spinal metastasis after HCC diagnosis was illustrated in Supplementary Table S2, and the median latency time to BM was six months.

**Figure 5:**
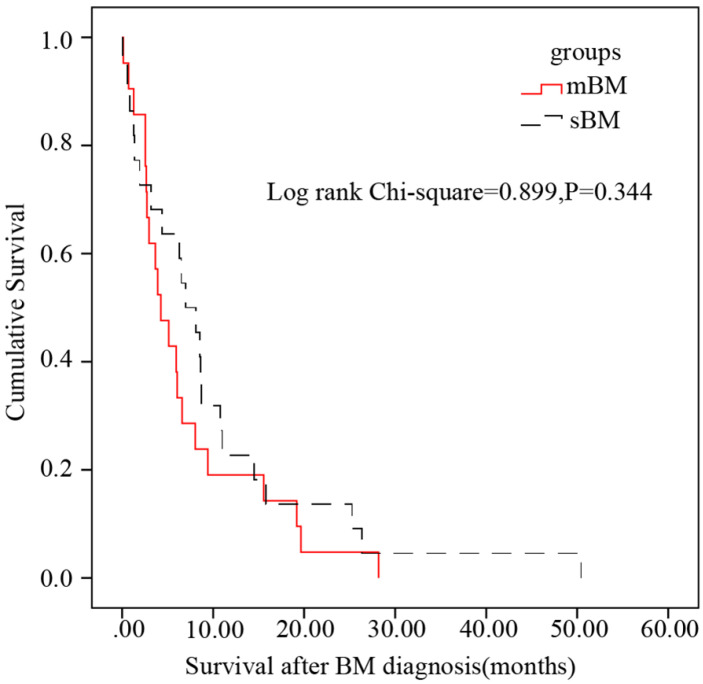
Kaplan-Meier survival after BM analysis in subsets of patients with BM. No statistical difference between patients with mBM and patients with sBM was found (P=0.344).

The median survival after BM was 4.27 months in HCC patients with mBM while 6.97 months in HCC patients with sBM. And after Log-Rank test, there was no statistical difference on survival after BM diagnosis between patients with mBM/sBM (*P*=0.344) (Figure 5).

### Prognostic analysis

Kaplan-Meier analysis and Cox regression model were used to identify prognostic factors for overall survival in HCC patients with BM. Besides the aforementioned involved variables, bone involvement numbers and bisphosphonates were included. A total of twenty-three variables were applied for univariate analysis, *P*-values less than 0.2 were included in the multivariable analysis. After univariate analysis, eight variables including intrahepatic tumor size, vascular tumor thrombus, serum ALP, HGB, WBC, PLT, TACE and bone involvement numbers were included for Cox regression analysis. Through overall survival analysis, vascular tumor thrombus was shown to be independent prognostic factor for BM patients with HR 3.114 (*P*=0.026), as shown in Table 5. Compared with the patients without vascular tumor thrombus, the patients with vascular tumor thrombus suggested the worse prognosis (Figure S3).

## Discussion

With the development of diagnosis and treatment technology, the survival of HCC patients has been improved. The incidence of BM in HCC has been increasing 9. The incidence of BM in HCC was reported to increase from 4.5% during 1978-1987 to 12.9% during 1988-1997 9.

The process of BM in HCC was accepted as a multi-step process, including cancer cells detach from the primary site, blood vessels invasion, distal capillaries migration and attachment into the bone, extravasate, blood supply recruitment and adjacent tissues invasion 10.

Different managements were given to HCC patients with sBM and mBM. Since BM was a significant restraint on the survival of HCC patients, the exclusion of sBM should be performed once HCC was diagnosed. Prognostic estimation should be firstly performed, and bone target therapy should be then considered once sBM was diagnosed in HCC patients 11. Currently, there has been no widely accepted BM screening strategy in HCC, early BM diagnosis can significantly improve the quality of life. Thus, the prediction on metastatic site and BM occurrence time in HCC patients with mBM are warrant. The present study gave the reference on both the specific metastatic site and time of mBM occurrence in HCC.

Patients with BM were usually with poor prognosis. Early detection of BM is crucial for patients to receive timely treatment. Therefore, the identification of high-BM risk is important. Due to the rarity of skeletal involvement from HCC, few studies looking into risk factors for BM in HCC was previously performed. Some studies reported that the expression of connective tissue growth factor (CTGF), interleukin-11 (IL-11) 12, chemokine receptor CXCR4 13 and LncRNA34a 14 can be potentially valuable predictive biomarkers for BM in HCC. Based on the Surveillance, Epidemiology, and End Results (SEER) database, it was shown that gender, marital status, T stage, lymph node involvement, intrahepatic metastases, and extrahepatic metastases were predictors of BM in HCC 5. In this study, we found that serum HGB and ALP level, and presence of lymph node metastasis were significantly correlated with BM occurrence in HCC. Tumor size was reported to be positively correlated with distant metastasis in HCC, tumor size (>58 mm) was 5.7 times more likely to develop distant metastasis than that of tumor less than 30 mm, and 2.9 times than that of tumor size 30-58 mm 15.The grade malignancy of the primary tumor can be significantly affected by T grade. This can partially explain the potential mechanism of our results. As previously reported in breast cancer, the larger size of the primary tumor found, the higher risk of the metastasis developed 16. Decreased HGB level was found to be one of the risk factors for sBM. Previous study reported that the decreased HGB level was associated with the metastatic risk in prostate cancer, which was consistent with our results 17. The mechanism underlying HGB and sBM occurrence needs to be further studied.

Previous studies suggested that the most common BM site in HCC was the axial bone, which may be caused by the formation of portal hypertension and collateral networks throughout the vertebral vein system 18. In our study, the most common BM site was the trunk bone. A total of 231 bone metastatic sites were found, including the lumbar vertebrae (55 sites) and thoracic vertebrae (54 sites), followed by the ribs (51 sites). For the first time, based on the single center of HCC cohort, we summarized the site-time data on mBM in HCC. Our results can potentially guide the clinical management and BM screening in HCC.

BM was a poor prognostic factor of patients with HCC, and the overall survival of patients with BM is worse than that of patients without BM. And the prognosis of BM patients can be even worse when the patients be diagnosed with vascular tumor thrombus. Previous studies showed that factors associated with the prognosis of HCC patients with BM were radiotherapy, chemotherapy and lung metastasis 19. Oral sorafenib in patients with extrahepatic metastasis can significantly improve the survival 20.

Since the study was a retrospective study, there were some limitations in the present study. Child-pugh classification, KPS score, and the degree of pathological grade of HCC were not collected in our dataset. Being lack of the external cohort, we were not able to perform external validation. In addition, incomplete follow-up data due to patient contact changes or poor adherence can affect the reliability of the data to some extent and we will also further expand the sample size to reduce bias.

## Conclusion

The incidence of sBM in HCC was 1.74% at initial diagnosis. The median latency time from HCC diagnosis to mBM was six months. Among HCC patients, lymph node metastasis, serum ALP >150 U/L, and HGB <110 g/L were correlated with sBM occurrence. Male gender, AB blood type, presence of extraosseous metastases, HGB <110 g/L and PLT <100×10^9^/L were associated with mBM occurrence. Among HCC patients with BM, the most common metastatic site was trunk, followed by upper limb bone, lower limb and skull. Such factors and characteristics can be potentially used for individualized BM screening and early diagnosis. A poor prognosis (median survival 8.61 months) was reported in our study. HCC patients with mBM showed better median survival than the patients with sBM. Vascular thrombosis was found to be correlated with the significant worse prognosis.

## Supplementary Material

Supplementary figures and tables.

## Figures and Tables

**Table 1:** The population distribution of HCC patients with BM and non-BM

Variable	Overall, N=391	Non-BM, N=263	BM, N=128	sBM, N=77	mBM, N=51
Male gender	339/391	225/263	114/128	66/77	48/51
Age at diagnosis, ≤60 years	251/391	168/263	83/128	52/77	31/51
**ABO blood type**					
A	101/385	71/263	30/122	18/76	12/46
B	106/385	77/263	29/122	24/76	5/46
AB	65/385	38/263	27/122	9/76	18/46
O	113/385	77/263	36/122	25/76	11/46
Smoking history	161/391	107/263	54/128	38/77	16/51
Alcohol-drinking history	110/391	81/263	29/128	18/77	11/51
Family history of cancer	75/391	52/263	23/128	11/77	12/51
Antiviral history	54/391	41/263	13/128	5/77	8/51
Intrahepatic tumor numbers, single	225/385	170/263	55/122	33/76	22/46
Intrahepatic tumor size, ≤5 cm	203/362	154/244	49/118	30/72	19/46
Vascular tumor thrombus	132/385	98/263	34/122	21/76	13/46
Intrahepatic metastasis	30/391	20/263	10/128	6/77	4/51
Extraosseous metastases	53/391	26/263	27/128	15/77	12/51
Lymph node metastasis	53/391	19/263	34/128	24/77	10/51
AFP, ≥25 ng/ml	216/385	140/263	76/122	46/76	30/46
ALP, >150 U/L	135/376	74/260	61/116	42/74	19/42
HBsAg, positive	289/385	200/263	89/122	54/76	35/46
**HGB, (g/L)**					
high (>160)	54/380	44/259	10/121	4/75	6/46
low (<110)	24/380	7/259	17/121	10/75	7/46
normal (110-160)	302/380	208/259	94/121	61/75	33/46
**WBC, (10^9^/L)**					
high (>10)	30/380	13/259	17/121	13/75	4/46
low (<4)	69/380	49/259	20/121	9/75	11/46
normal (4~10)	282/380	197/259	85/121	53/75	32/46
**PLT, (10^9^/L)**					
high (>300)	32/380	16/259	16/121	9/75	7/46
low (<100)	60/380	38/259	22/121	11/75	11/46
normal (100~300)	289/380	205/259	84/121	55/75	29/46

**Table 2:** The baseline characteristics comparison of HCC patients with BM and non-BM and risk factor analysis of BM

Variable	Chi-Squared Test	Univariate analysis		Multivariate analysis	
	*P*	*P*	OR	*P*	OR (95% CI)
Male gender	0.337	0.339	1.375		
Age at diagnosis, ≤60 years	0.852	0.852	1.043		
**ABO blood type**	0.199				
A		0.733	0.904		
B		0.467	0.806		
AB		0.195	1.520		
O			1(reference)		
Smoking history	0.777	0.777	1.064		
Alcohol-drinking history	0.093	0.094	0.658	0.027	0.512(0.282-0.927)
Family history of cancer	0.671	0.671	0.889		
Antiviral history	0.144	0.147	0.612		
Intrahepatic tumor numbers, single	<0.001	<0.001	0.449	0.008	0.491(0.291-0.828)
Intrahepatic tumor size, ≤5 cm	0.001	<0.001	0.415	0.023	0.530(0.307-0.915)
Vascular tumor thrombus	0.001	0.072	0.651		
Intrahepatic metastasis	0.942	0.942	1.030		
Extraosseous metastases	0.002	0.003	2.437		
Lymph node metastasis	<0.001	<0.001	4.645	<0.001	4.073(1.900-8.733)
AFP, ≥25 ng/ml	0.01	0.096	1.452		
ALP, >150 U/L	<0.001	<0.001	2.788	0.004	2.271(1.308-3.944)
HBsAg, positive	0.002	0.514	0.850		
HGB (g/L)	<0.001				
high (>160)		0.064	0.503	0.219	0.586(0.250-1.375)
low (<110)		<0.001	5.374	0.004	5.026(1.671-15.120)
normal (110-160)			1(reference)		1(reference)
WBC (10^9^/L)	0.006				
high (>10)		0.005	3.031		
low (<4)		0.851	0.946		
normal (4~10)			1(reference)		
PLT (10^9^/L)	0.019				
high (>300)		0.018	2.440		
low (<100)		0.245	1.413		
normal (100~300)			1(reference)		

**Table 3:** Multivariate analysis for sBM compared with non-BM

Variable	Univariate analysis		Multivariate analysis		
	*P*	OR	*P*	OR	95% CI
Intrahepatic tumor size, ≤5 cm	0.001	0.417	0.031	0.486	0.252-0.936
lymph node metastasis	<0.001	5.815	0.001	4.332	1.831-10.249
ALP >150 U/L	<0.001	3.299	0.003	2.666	1.391-5.111
**HGB (g/L)**					
low (<110)	0.002	4.871	0.040	3.909	1.067-14.324

**Table 4:** Multivariate analysis for mBM compared with non-BM

Variable	Univariate analysis		Multivariate analysis		
	*P*	OR	*P*	OR	95% CI
Male gender	0.109	2.702	0.012	12.132	1.744-84.401
**ABO blood type**					
AB	0.005	3.316	0.024	3.239	1.167-8.989
Alcohol-drinking history	0.188	0.618	0.027	0.354	0.142-0.886
Intrahepatic tumor numberds,single	0.032	0.501	0.046	0.449	0.204-0.987
Extraosseous metastases	0.008	2.805	0.016	3.769	1.284-11.060
**HGB (g/L)**					
low (<110)	0.001	6.303	<0.001	17.627	3.787-82.041
**PLT (10^9^/L)**					
low (<100)	0.070	2.046	0.039	2.893	1.053-7.949

**Table 5:** Univariate and multivariate analyses of prognostic factors in BM groups

Variable	Non-BM, N=40	BM, N=43	Univariate analysis		Multivariable analysis		
			χ^2^	*P*	*P*	HR	95% CI
Intrahepatic tumor size, ≤5cm	19/37	16/41	4.213	0.122	0.778	0.896	0.417-1.925
Vascular tumor thrombus	17/40	13/42	4.775	0.092	0.026	3.114	1.144-8.475
ALP >150 U/L	18/40	23/40	7.126	0.028	0.167	2.077	0.737-5.849
HBsAg, positive	28/40	29/42	2.800	0.247			
HGB (g/L)							
high (>160)	5/40	2/41	2.882	0.090	0.770	0.697	0.062-7.824
low (<110)	2/40	7/41	0.087	0.768	0.400	1.536	0.565-4.176
normal (110~160)	33/40	32/41	0.799	0.371		1(reference)	
**WBC (10^9^/L)**							
high (>10)	4/40	6/41	2.377	0.123	0.205	0.420	0.110-1.605
low (<4)	6/40	7/41	4.173	0.030	0.587	1.527	0.331-7.057
normal (4~10)	30/40	28/41	7.043	0.008		1(reference)	
**PLT (10^9^/L)**							
high (>300)	6/40	6/41	4.474	0.034	0.136	0.436	0.147-1.298
low (<100)	6/40	10/41	5.328	0.021	0.488	0.613	0.153-2.446
normal (100~300)	28/40	25/41	5.106	0.024		1(reference)	
Primary tumor surgery	26/40	7/41	<0.001	0.990			
TACE	7/40	5/43	1.941	0.164	0.412	1.573	0.533-4.642
Bone involvement, solitary	16/42	16/42	4.832	0.089	0.375	0.674	0.282-1.611

## Abbreviations

HCC: hepatocellular carcinoma
BM: bone metastasis
sBM: synchronous bone metastasis
mBM: metachronous bone metastasis
ALP: alkaline phosphatase
HGB: hemoglobin
PLT: platelet
WBC: white blood cell
TACE: Transarterial chemoembolization
AFP: alphafetoprotein
