# Supplementary material for: The homogeneity and heterogeneity of occurrence, characteristics, and prognosis in hepatocellular carcinoma patients with synchronous and metachronous bone metastasis
Source: J Cancer. 2022 Jan 1;13(2):393–400. doi: 10.7150/jca.65308 (PMC8771510; doi:10.7150/jca.65308)
Supplement: Supplementary file 1 — Supplementary figures and tables. [file jcav13p0393s1.pdf]

Supplementary material

Supplementary figures

Figure S1. Forest plot of relative factors associated with sBM occurrence. OR (odds ratio), 95%CI (confidence interval).

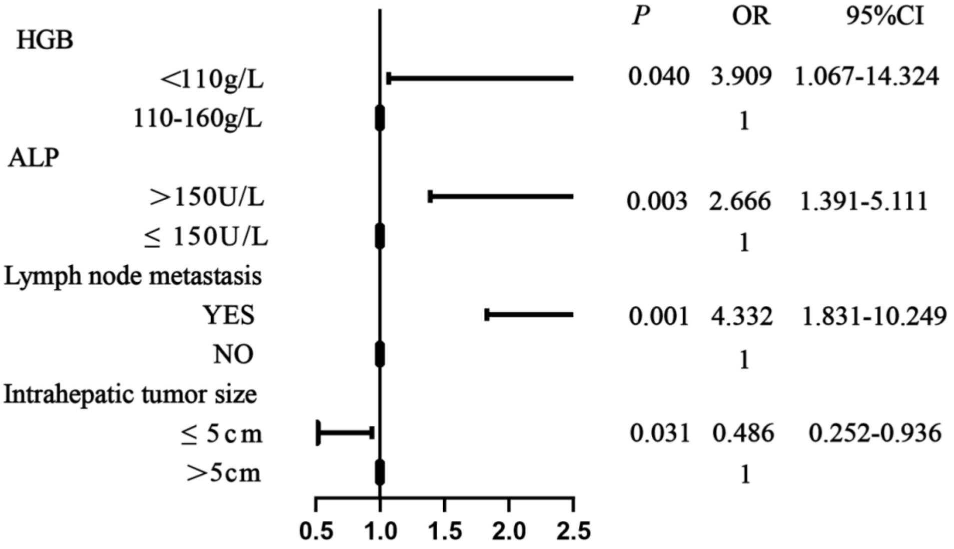

Figure S2. Forest plot of relative factors associated with mBM occurrence. OR (odds ratio), 95%CI (confidence interval).

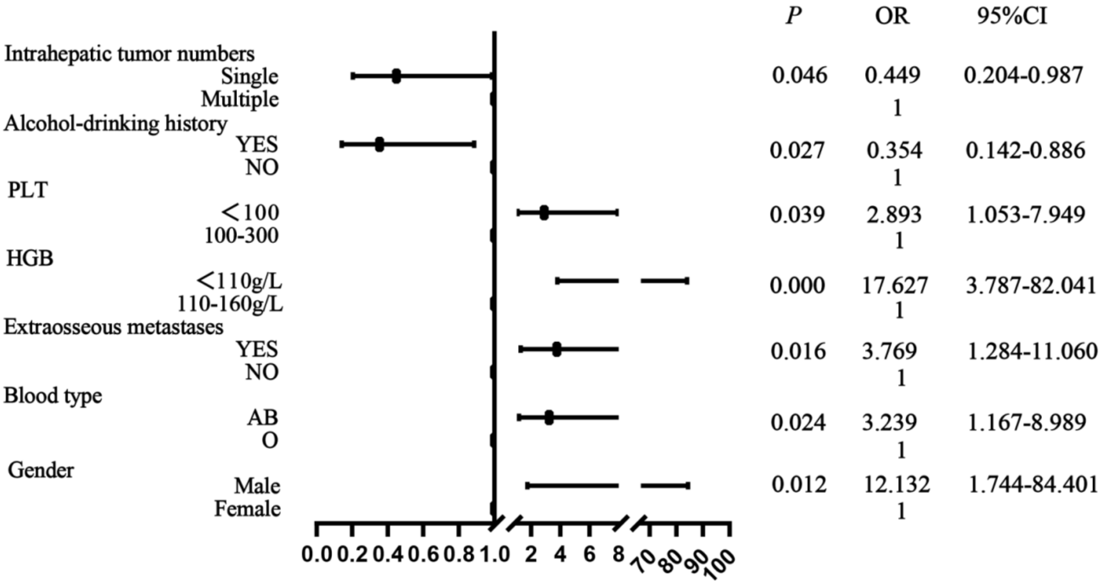

Figure S3. Overall survival analysis of HCC patients with BM according to vascular tumor thrombus.

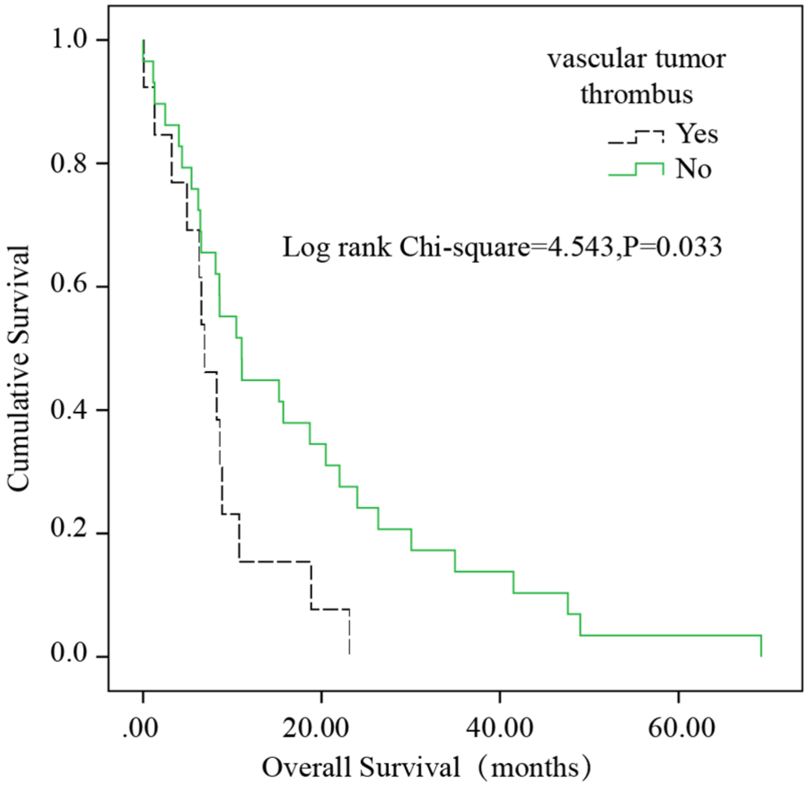

Supplementary tables

Table S1. The localization of extraosseous metastatic sites.

Localization	Number of extraosseous	Percentage
**Lung**	18	53.0%
**Adrenal gland**	3	8.8%
**Abdominal wall**	3	8.8%
**Peritoneum and retroperitoneum**	5	14.7%
**Chest wall**	2	5.9%
**Spleen**	2	5.9%
**Brain**	1	2.9%
**Total**	34	100%

Table S2. The distribution of spinal metastasis after HCC diagnosis in mBM group.

**Spine**	**Total occurrences**	**Time after HCC diagnosis(months)**			
	**N=64**	**6**	**6-12**	**12-24**	**>24**
**Cervical**	5	1（20.0%）	1（20.0%）	2（40.0%）	1(20.0%）
**Thoracic**	23	16（69.6%）	3（13.0%）	1（4.4%）	3(13.0%)
**Lumbar**	21	13（61.9%）	2（9.5%）	3（14.3%）	3(14.3%)
**Sacrum**	15	10（66.7%）	2（13.3%）	2（13.3%）	1(6.7%)
